# Supplementary material for: Developmental trajectories of EEG aperiodic and periodic power: Implications for understanding the timing of thalamocortical development during infancy
Source: Res Sq. 2023 Sep 18:rs.3.rs-3215728. Preprint. [Version 1] doi: 10.21203/rs.3.rs-3215728/v1 (PMC10543027; doi:10.21203/rs.3.rs-3215728/v1)

Supplemental Figure 1 - Electrode layout: (A) 128-channel Hydrocel Geodesic Sensor Net. (B) 64-channel Geodesic Sensor Net. Pink circles denote 10-20 electrodes, and blue circles denote the additional electrodes included in ICA and MARA steps of pre-processing. (C), (D) Electrodes averaged for frontal (yellow), central (blue), temporal (orange), and posterior (green) regions of interest.

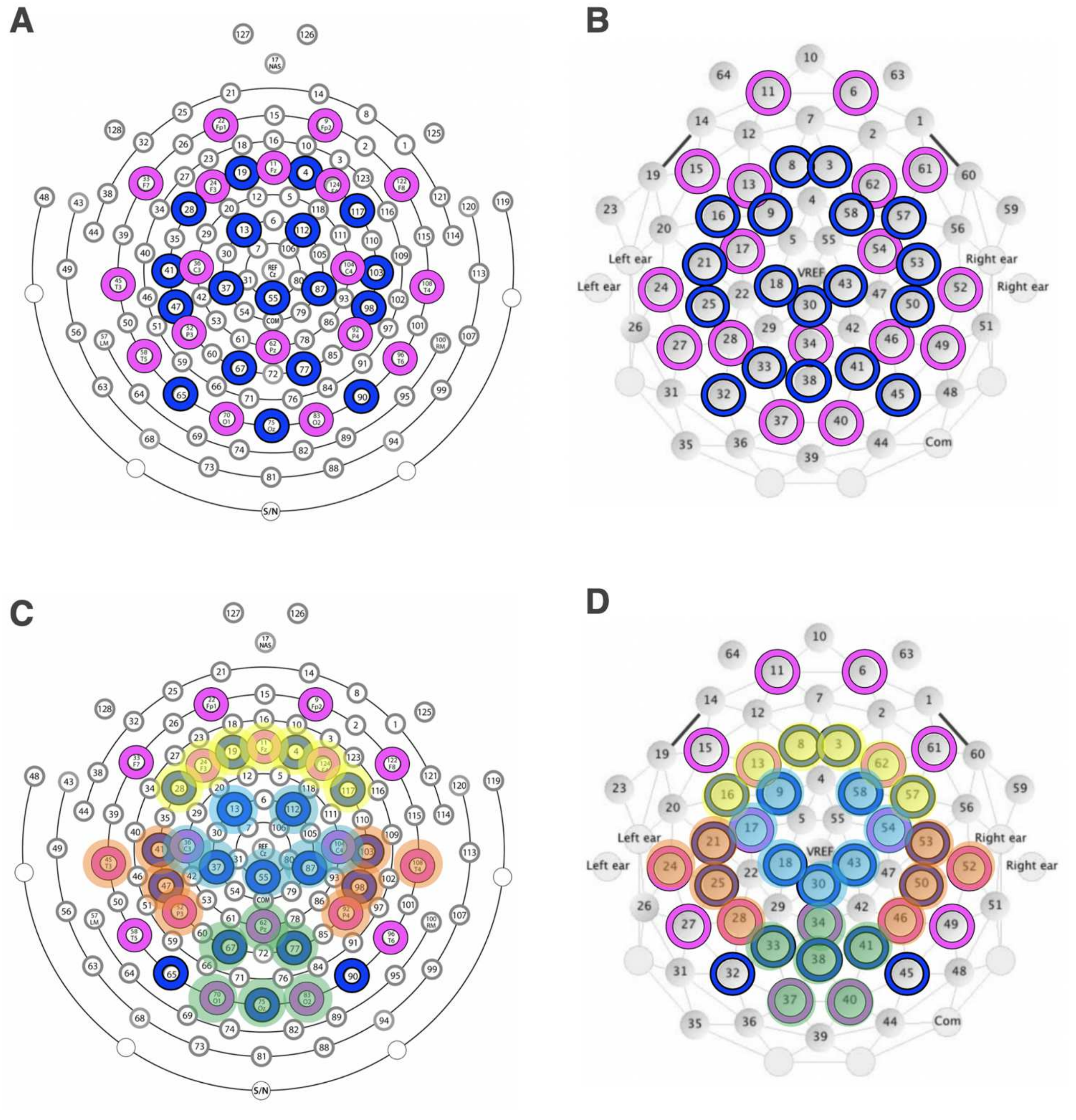

Supplemental Figure 2: Individual plots of the periodic spectrum averaged across the whole ROI for each age bin.

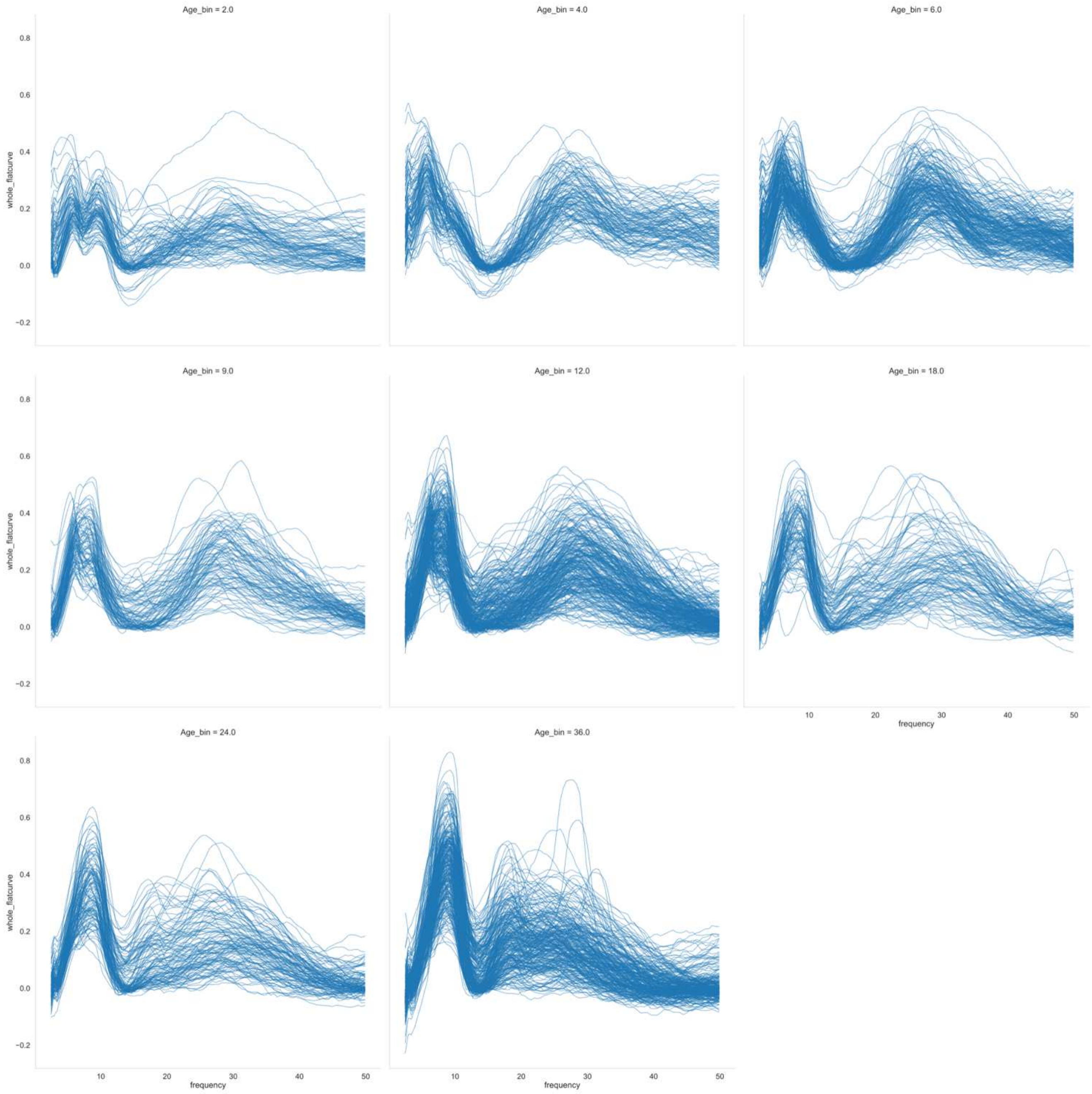

Supplemental Figure 3: GAMMs modeled trajectories of 8 power measures, with ROI, study, smoothed age, and sex as predictor terms.

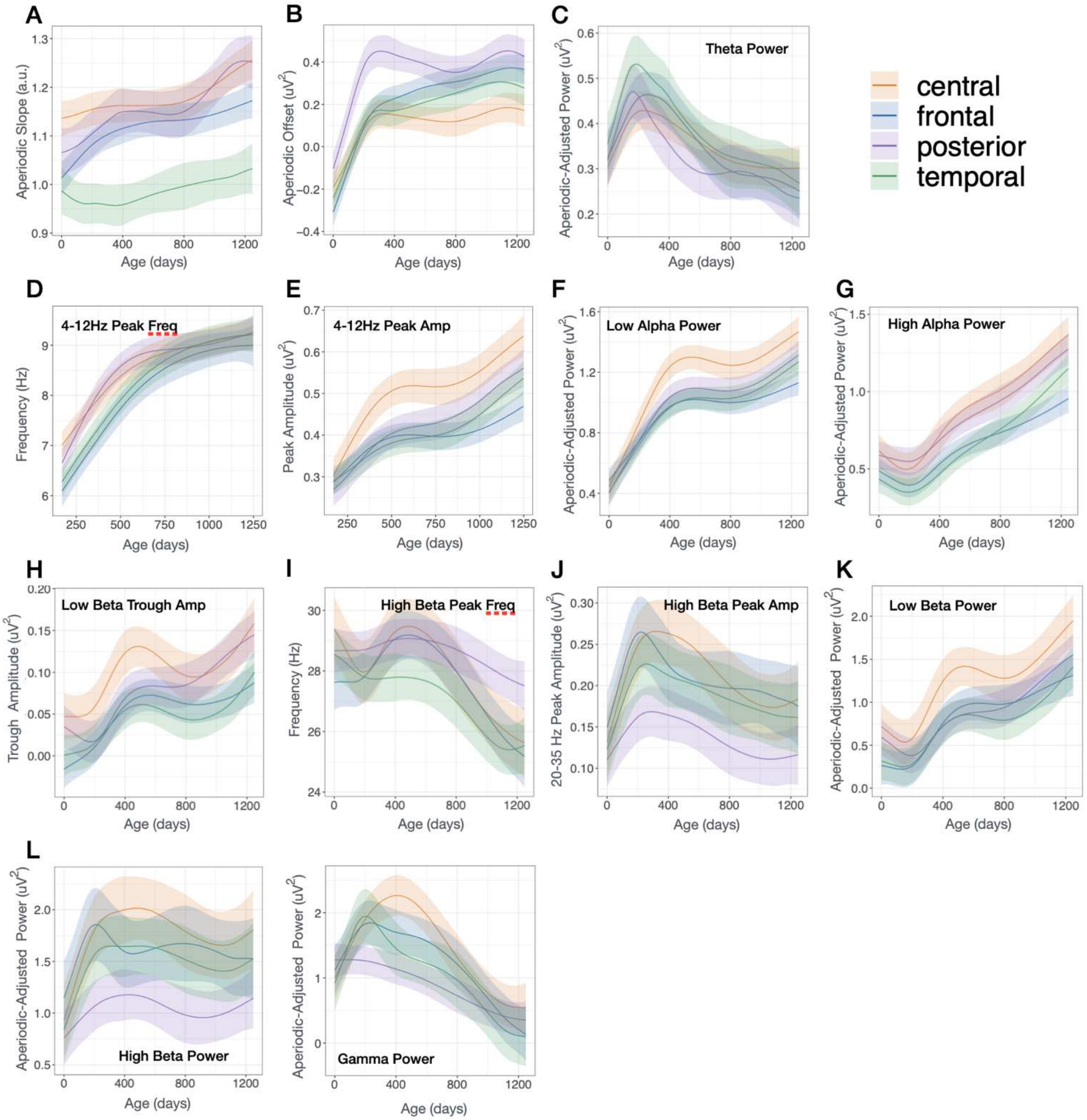

Supplemental Figure 4: For each age bin the average FOOOF modeled spectra (green) across participants in each age bin is graphed along with the averaged original power spectrum (red), and averaged FOOOF estimated aperiodic spectrum (blue). (A) Unedited FOOOF estimates. (B) Modified FOOOF estimates. (C) Comparison of squared error across frequencies of unedited (orange) and modified (blue) FOOOF.

A. Unedited FOOOF model estimates - Original Spectrum - FOOOF modeled Spectrum - Aperiodic Fit

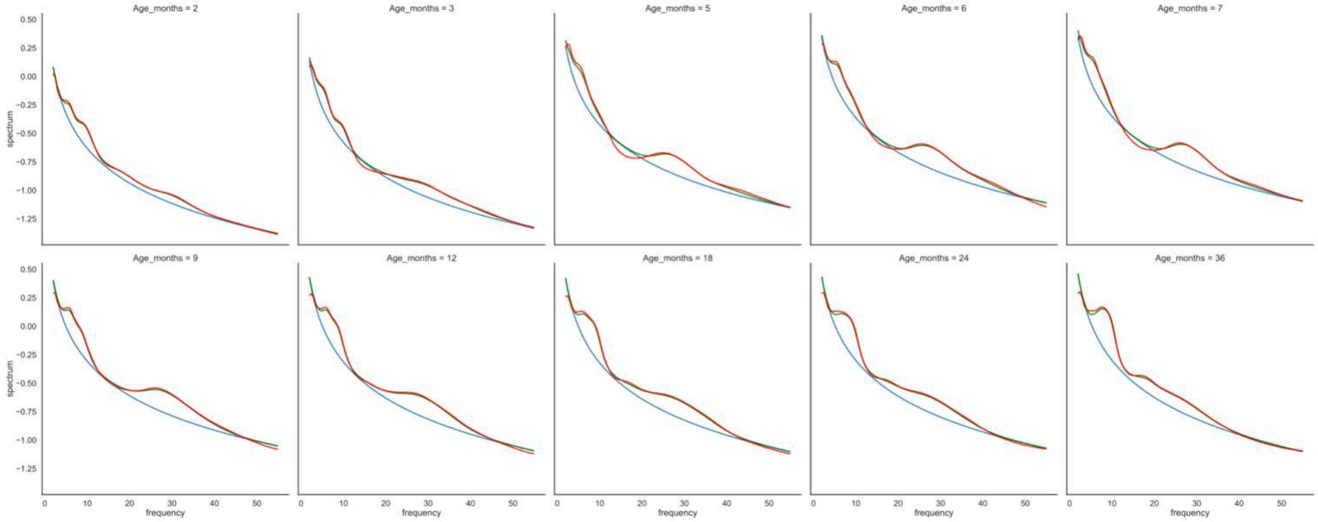

B. Modified FOOOF model estimates - Original Spectrum - FOOOF modeled Spectrum - Aperiodic Fit

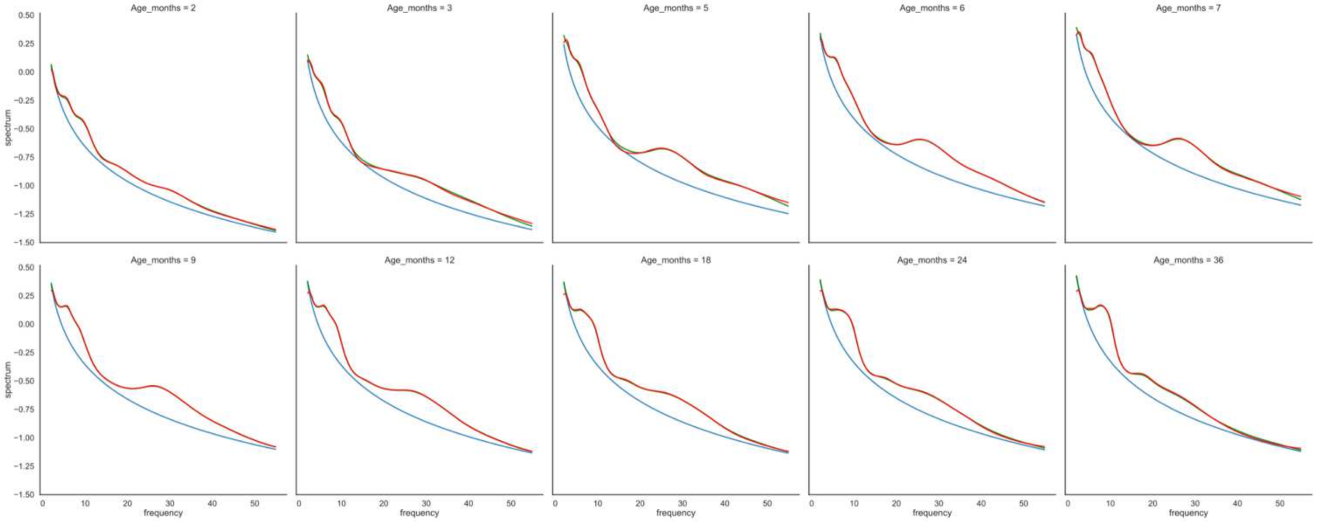

C. Original vs Edited FOOOF error

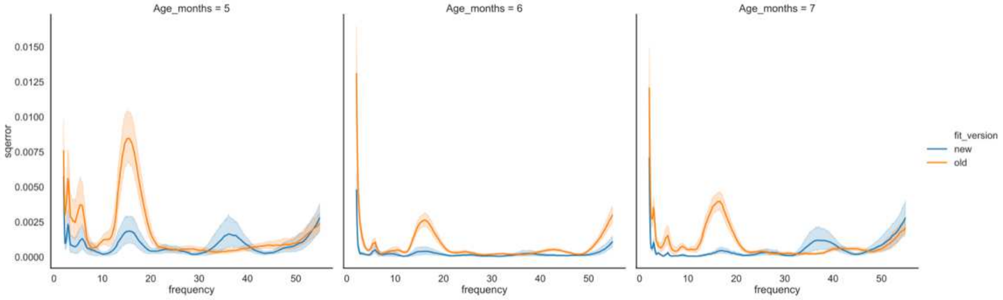

Supplement: Supplement 1 [file NIHPPrs3215728v1-supplement-1.pdf]
